# What happens when people develop dementia whilst working? An exploratory multiple case study

**DOI:** 10.1080/17482631.2023.2176278

**Published:** 2023-02-17

**Authors:** Louise Nygard, Ann-Charlotte Nedlund, Anna Mäki Petäjä Leinonen, Arlene Astell, Jennifer Boger, Mervi Issakainen, Ann-Louise Engvall, Birgit Heuchemer, Lena Rosenberg, Charlotta Ryd

**Affiliations:** a Division of Occupational Therapy, Department of Neurobiology, Care Sciences and Society, NVS, Karolinska Institutet, Stockholm, Sweden; b Division Society and Health, Department of Health, Medicine and Caring Sciences, Linköping University, Linköping, Sweden; c Faculty of Social Sciences and Business Studies, Law School, University of Eastern Finland, Joensuu; d Department of Social Sciences, Faculty of Social Sciences and Business Studies, Kuopio; e Department of Occupational Sciences & Occupational Therapy & Department of Psychiatry, University of Toronto, Toronto, Canada; f Systems Design Engineering, University of Waterloo, Waterloo, Canada; g School of Health and Welfare, Jönköping University, Jönköping, Sweden; h Stockholm Gerontology Research Center, Stockholm, Sweden

**Keywords:** Accessibility, accommodation, agency, Alzheimer’s disease, case study, disclosure, early onset, employment, subjective experiences, stigma

## Abstract

**Purpose:**

This study is an in-depth exploration of the unfolding experiences of five persons who developed dementia while still in paid work/employment, and of their significant others. Namely, we explore how they experienced the actions and decisions taken with respect to work, and what the consequences meant to them.

**Methods:**

A qualitative longitudinal case study design with multiple cases was used, including five participants with dementia and significant others of their choice. Interviews were undertaken longitudinally and analysed with the Formal Data-Structure Analysis approach.

**Results:**

The joint analysis resulted in two intertwined themes: 1) The significance and consequences of a dementia diagnosis: a double-edged trigger, and 2) Sensemaking and agency. The prevalent images of what dementia is, who can/cannot get it and what it will bring, were revealed as the critical aspects. Having the opportunity to make sense of what has happened and participate in decision-making, contributed decisively to the participants’ experiences.

**Conclusions:**

Findings illustrate how a dementia diagnosis is alien in work-life, but once diagnosed, it may trigger self-fulfiling expectations based upon stereotypical understanding of dementia. A shift is needed from a deficit-focused perspective, to viewing people with dementia as citizens capable of agency.

## Introduction

Current predictions suggest that by 2030, 78 million people worldwide will be living with dementia, almost double the number in 2015 (Gauthier et al., 2021). Dementia is an irreversible neurological disorder, diagnosed based on changes in cognitive ability, such as memory and planning, with accompanying changes in behaviour. Advances in diagnosis meant that dementia can be identified earlier and at younger ages (Dubois et al., 2014), even before symptoms manifest in everyday life. This is sometimes referred to as prodromal dementia.

Increasing understanding and awareness of the prevalence of dementia means that it is not just an issue for medicine and care; it also concerns wider society, including the workplace (Cahill, 2020; Egdell, Stavert & McGregor, 2017). Interest in dementia and working life is increasing, framed mainly as an issue for people with young onset dementia, i.e., below the age of 65 years (Evans, 2019; Ikeuchi et al., 2020; Roach, 2017). At the same time, retirement ages are rising across high-income countries, and people are being encouraged to work beyond accepted working ages, i.e., 65 years (OECD, 2021). Thus, employees of varying ages are experiencing early symptoms of dementia, and their symptoms may appear in a wide range of presentations. Moreover, there are almost infinite variations of work roles and different types of employment, which are more or less able to accommodate dementia.

Alongside interest in dementia in the workplace, a rights-based approach to dementia policy and planning, emphasizing agency, self-determination, participation, equity, and choice has emerged across Western countries (Cahill, 2020). To enact this a greater understanding of the lived experience of dementia is required. Applying a `citizenship lens´ to the lived experiences of dementia provides a language for a rights-based understanding of these experiences. The citizenship lens also emphasizes the importance of an individual’s social position and agency (O’connor & Nedlund, 2016) while further challenging legal directions and practices (Nedlund & Taghizadeh Larsson, 2016). In recent years, dementia has been recognized as a cause of disability, implying an “opportunity to campaign for rights, advocate for change and be covered by legislation promoting human rights” (Alzheimer Europe, 2017, p. 39). Recognizing people with dementia as disabled also means that national provisions should apply equally to them as to people with other disabilities. A rights-based approach recognizes the rights and capability of people with dementia to express their experiences and identify the important issues in their own lives (Alzheimer Europe, 2017). Acknowledging the complexity of developing dementia whilst in the workforce, the present study is an in-depth exploration of the unfolding experiences of five persons in Sweden who developed dementia while still in paid work/employment, and of their significant others. A qualitative, longitudinal, multiple case approach was used to explore the experiences of the persons with dementia in their work context and those of significant others in their lives (i.e., persons from family and/or work).

## Literature review

Research has mostly taken either the perspective of employees with dementia (Andrew et al., 2018; Chaplin & Davidson, 2016; Evans, 2019; Ikeuchi et al., 2020; McCulloch et al., 2016), or of employers of people with dementia (Cox & Pardasani, 2013; Stavert et al., 2018; Egdell et al., 2019), with just a few studies considering both (Ritchie et al., 2018, 2020; Öhman et al., 2001). It has consistently been reported that symptoms of dementia are usually been first noticed at work, are difficult to define, and cause uncertainty and stress for the employees as well as the employers (Chaplin & Davidson, 2016; Evans, 2019; Ikeuchi et al., 2020; McCulloch et al., 2016; Roach, 2017; Silvaggi et al., 2020; Öhman et al., 2001). Diagnostic delay is common and not surprising as statistically, dementia is not the most likely cause of subtle cognitive decline in working-aged persons. The classic assessment tools used to diagnose dementia are not sensitive enough to capture early signs of younger onset dementia that commonly differ from the common development of Alzheimer's disease, AD (Silvaggi et al., 2020).

Once people obtain a diagnosis, there may be long delays before disclosure due to the stigma associated with dementia (Bhatt, 2020). This is especially so in the workplace where a ‘geriatric´ diagnosis, such as dementia, is not commonly seen or expected. The issue of continued employability and work is usually related solely to the capability of the person with dementia (e.g., Ritchie et al., 2020; Silvaggi et al., 2020). However, this approach has been criticized as being far too limited, ignoring the complexity of paid employment and the potential of social and physical environments to support continued work (Ritchie et al., 2020). Despite the disabling consequences of dementia, many studies argue that continued employment post diagnosis, would be preferable, because work is such an important part of life, especially for people of working age (Ritchie et al., 2015; Roach, 2017). For society at large, continued work promotes social inclusion and may impact positively on societal costs (Silvaggi et al., 2020).

For someone who develops dementia, continued employment is likely to require adjustments or accommodations at work, which can be difficult to support (Ritchie et al., 2018; Roach & Drummon, 2014; Stavert et al., 2018; Öhman et al., 2001). To facilitate continued employment, a person-centred approach, including collaboration, guidance, and training for employers on legal and human rights, has been suggested (Andrew et al., 2018; Issakainen et al., 2021; Ritchie et al., 2018; Stavert et al., 2018). Specifically, the *process* of making adjustments, e.g., changing work schedules, simplifying routines, using technology for reminders, has been identified as a key theme requiring urgent attention (Thomson et al., 2019). Many studies have identified lack of awareness about dementia among employers as a possible cause of disability or changes in performance (e.g., Egdell et al., 2018; Egdell et al., 2019; McCulloch et al., 2016; Ritchie et al., 2015; Stavert et al., 2018). Health care professionals also need to consider the implications for employment when a person is diagnosed with dementia, such as discussing the potential for them to keep working and identifying accommodations to keep them in the workforce (Andrew et al., 2018).

The current literature on dementia and work/employment highlights the need for increased awareness that dementia might be present in the workforce and how it may appear, e.g., in change in performance. Additionally, there is limited knowledge about how to handle situations with employees who develop dementia, at all levels from individual employers to legal systems, government policies, and social welfare. While it has been underscored that continued work might be preferrable, most studies report that receiving a diagnosis of dementia means immediately leaving work (either because the employee chooses to leave or the employer terminates them) without much effort to understand the situation, explore possible alternatives, or make adjustments (Evans, 2019; McCulloch et al., 2016; Roach & Drummon, 2014; Thomson et al., 2019). A recent study in Finland found that people with dementia may not be given the opportunity to participate in making decisions about continuing or leaving employment (Issakainen et al., 2021). This exemplifies the challenge of implementing a rights-based approach enacting each person’s right to legal capacity, i.e., the right of a person with dementia to get support in decision-making (Cahill, 2020).

Despite these recent studies, understanding of what happens when a person develops dementia while still working is limited. Using a multiple case study approach, our aim was to perform an in-depth exploration of the unfolding experiences of five persons who developed dementia while still in paid work/employment and their significant others from work, health care and family. Specifically, we want to examine how they experienced the actions and decisions taken with respect to work and what the consequences meant to the individuals with dementia and their significant others.

## The Swedish context

As work life is shaped and regulated by a country’s legislation, this aspect of the context will have a profound impact for a person who develops dementia while still employed, and the process of leaving/ending work may look different in different countries. Legislation is also likely to influence the importance and meaning of continued work for the employee. Many international human rights instruments such as the UN Convention on the Rights of the Persons with Disabilities (UNCRPD) bind countries worldwide. According to Article 27 of the UNCRPD, it is the right of persons with disabilities to work on an equal basis with others (UNCRPD, 2022). This includes the right to the opportunity to gain a living by work that is freely chosen or accepted in a labour market plus a work environment that is open, inclusive, and accessible to persons with disabilities. UNCRPD states that parties shall safeguard and promote the realization of the right to work by taking appropriate steps, including those who acquire a disability during employment.

In Sweden, the legislation supports continued work for people with disabilities. The employer is responsible for adapting the workplace to meet the employee’s needs. Individually targeted measures should be taken by the employer together with occupational health and rehabilitation services (Karlsson et al., 2014). While the right to self-determination for all adults is emphasized by Swedish laws and regulations, there are no regulations to guide supported decision-making for people with disabilities (Nedlund & Taghizadeh Larsson, 2016). Recognizing people with dementia as disabled also means that national provisions should apply equally to them as to people with other disabilities. The Swedish Social Insurance Agency (SSIA) is responsible for providing financial security in the event of disability or illness. Severe dementia is listed as a serious illness that entitles the person to sickness benefit. SSIA also has a legal obligation to coordinate activities and contacts if return-to-work rehabilitation is required (SSIA, 2021). However, it is up to each employer to seek out information and develop a plan of action on a case-by-case basis with their employees. If continued work is impossible, the employee can apply for sickness compensation. If this is granted, the employer is no longer responsible for rehabilitation or adjustments at work and can end the employment after notifying the employee in writing.

## Methods

### Study design

This research was designed as a qualitative longitudinal case study with multiple cases (Merriam, 1998). Each case consists of a person with dementia at the centre and significant others involved in each case (e.g., family, friends, work-related persons or persons from memory investigation or rehab). We chose this approach as recommended by Merriam (1998) because the topic requires investigation within its real-life context, were we expected the participants’ experiences and the context to be interwoven and without clear boundaries. Our point of departure was the individual experiences of working life of the person living with dementia, the changes happening and the decisions and measures that were taken at the onset of the cognitive decline (by the persons with dementia, their workplace, and their significant others), including how these measures contributed to their situation and well-being in what we call “their journey”. Our specific interest was to gain an understanding of the events that occurred and the actions that were taken, how events and actions were experienced, what they led to, and how they contributed to their unfolding journeys (Rosenberg & Johansson, 2013). The study is part of a larger research project initiated in 2018: “Dementia or MCI @ work”, which is an international, multidisciplinary research project under the Joint Program Initiative umbrella of More Years Better Lives (MYBL) (http://www.jp-demographic.eu/calls/projects/).

### Inclusion criteria, recruitment, and ethics

*Recruitment of participants with dementia*: The criteria set for inclusion were—based on self-reports—that each participant with dementia: i/had received a diagnosis of MCI or dementia or were still in the process of memory investigation but with suspected dementia, ii/was working; employed or self-employed, or had been engaged in paid work within the latest six months, iii/was or had been on sick-leave (full or part time) no more than six months, iv/was 50–75 years old, and v/was capable, willing and interested to take part in the longitudinal data collection. Our hope was to achieve variation in the participants’ fields of work, as well types of employment, length of sick-leave, education, age, and sex. However, we soon discovered that finding participants who met the second criteria was a challenge, and variation in all criteria could not be achieved.

Recruitment was through a variety of settings where we thought it would be possible to find persons that met our inclusion criteria based on our clinical experience. Researchers presented the study at a unit for investigation of driving capacity, an out-patient unit for investigation of early cognitive impairment, and a voluntary group for people with young onset dementia. Staff at these facilities shared information about the study and supported people with dementia who were potentially interested in participation in the study. Two participants were recruited from the unit for investigation of driving capacity and the voluntary group, respectively, and one from the unit for investigation of early cognitive impairment. The demographics of the five included persons-three women and two men, aged between 53 and 71 years—are presented in Table I, together with details about the data collection and significant others included in each case. Recruitment took place as a process from December 2018 until August 2019, which meant that cases were in part overlapping and ongoing simultaneously, with the final interview in March 2021.

**Table I.** Demographics of participants with dementia, list of their significant others, and an overview of data collection parameters.

Case number and given name	Living situation	Work % and employment or time since work cessation at inclusion	Significant others (SOs) included in the case	Period of data collection	Total number of interviews
1 Simon	Co-habiting	0%, No employment One week	Wife Grown up son	December 2018 – November 2019	6 (4 with Simon, 2 with SOs)
2 Anna	Co-habiting	0%, No employment Six months	Former boss Grown up daughter Work leader	February 2019 – February 2020	6 (4 with Anna, 2 with SOs)
3 Lisa	Single	0%, Permanent employment Six months	Sister Work leader (rehab training) Social worker Former boss	March 2019 – November 2019	9 (6 with Lisa, 3 with SOs)
4 Maria	Co-habiting	40%, Permanent employment	Best friend and colleague	August 2019 – March 2021	6 (5 with Maria, 1 with SO)
5 Carl	Co-habiting	25%, Consultant	Work leader/employer	August 2019 – June 2020	5 (4 with Carl, 1 with SO)

*Recruitment of significant others*: To build a confident relationship based on trust with the participants with dementia, we left the decision about who to invite as significant others up to them. As we wanted to broaden the view, we encouraged them to think of significant others that were informed and engaged in their unfolding situation from a family perspective, at work and/or in health care. These persons were then invited as participants, but for the sake of clarity they are referred to as significant others in the text when relevant.

*Consent to participate*: All participants received both verbal and written information before they gave consent to take part in the study. Participants with dementia were repeatedly informed that they had the right to exit the study at any point without further justifications of such a decision. Approval was given by the regional ethical board (file number 2018/1313–31/5).

### Data collection and analysis

Qualitative, conversational interviews (Brinkmann & Kvale, 2018) were conducted one-to-one with the five participants with dementia and the significant others of their choice. In total, 33 interviews were conducted (see Table I). These most often took place in the participants’ homes, with a few with significant others in the researcher’s office. The intention was to follow each case until a new stability was achieved in relation to what happened vocationally. This led us to follow each case for around one year (see Table I), with the same researcher conducting all interviews within a given case. An interview guide with broad topics (Brinkmann & Kvale, 2018) was created to be open for each person’s evolving situation. In each case, the data collection was initiated by an interview where the participant with dementia spoke about what had happened at work and in everyday life from the time of their first subtle symptoms to when their dementia was diagnosed and continuing to their current situation. This was then elaborated more in depth in subsequent interviews over time. For some participants, it meant looking back at work-life and the cessation of work, and for others reflecting on the present. The interview topics focused on the people living with dementia’s experiences of the decisions and actions taken within their employment, and what the subsequent consequences meant to them. The interview topics for significant others were designed to elicit elaboration on what had happened at work and in everyday life before, during and after dementia was diagnosed, and the subsequent consequences. Each case varied in terms of the number of participants, from a minimum of one significant other to a maximum of four.

As recommended by Merriam (1998), data collection, transcription and analysis took place in parallel. For example, memos were recorded in connection to each interview as well as in relation to transcribing. Data were continuously discussed within the research team with the aim of identifying questions or topics that could give valuable information in subsequent interviews, or guide selection of additional significant other participants. This meant that the interviews in the latter cases were in part influenced by the earlier cases, reflecting our learning during the ongoing analysis.

The Formal Data-Structure Analysis (FDSA) approach (Borell et al., 2012; Gustavsson, 2000) was used to analyse the interviews. Epistemologically grounded within the hermeneutic tradition (Kinsella, 2006), this analytical approach offers the possibility to study meaning-making as experienced and enacted in peoples’ lives, by shifting between two interpretation interests: on the one hand, the *experience-near* level (understanding individual persons’ experiences), and on the other hand, the *experience-distant* level (understanding the phenomenon on a more generalized level). In the present research, the analytical process—from the start of data collection and onwards—shifted between focusing on the empirical cases and their experiences, and the conceptual level, based upon theoretical or conceptual knowledge of e.g., how symptoms of dementia might influence work ability. This also led us to first present the five cases in experience-near detail, before comparing and contrasting them in a joint analysis to facilitate external validity (Merriam, 1998). This resulted in themes with interpretations as well as a synthesis of the themes.

When data collection approached the end, hands-on analysis started with a thorough line-by-line reading of all the interviews within each case. Meaning units were identified and given a code or short descriptive text suggesting the induced meaning, although still on the experience-near level. From this first thorough reading, we discovered that there were extensive differences between cases in what had happened and in the actions that had been taken at work, as well as in how our participants experienced the consequences. In the continued comparison of the cases, two features stood out that could give some clues to understanding the participants with dementia’s experiences; first, three of the five participants with dementia had some kind of work adaptations, but the adaptations were organized based upon different rationales and occurring at different time points in their journey in relation to being diagnosed. This led us to the second feature, which was how the first subtle signs of the problems were understood and dealt with at work. These two features offered insights into the processes and actions taken by health care and employers, as told by the participants with dementia and their significant others, providing a description of the conditions in their journeys. Our analysis continued focusing on the participants’ experiences of navigating within these conditions, and we discovered that the participants’ experiences of their predicament and the possibilities they were given at work differed profoundly. The immediate understanding was that the extent to which the person with dementia had a say in decisions influenced how they experienced the chain of events; namely, the person with dementia’s participation in decisions regarding their employment impacted their perceived experience.

According to the FDSA analytical approach, interpretations emerging from data are to be critically examined (Gustavsson, 2000). The next step then was to go back to the interviews and look for data that might speak against our immediate understanding, and for other aspects potentially leading to a different understanding of the experiences in each case. Eventually, the process of going back and forth between data and emerging findings, both within and across cases, led to identification of two inter-related themes (presented below). These two themes arose from our understanding of what contributed to the `journey´ of each participant with dementia, and what it meant to them. This iterative process also involved repeated discussions among the research team plus parallel use of the literature, in the search for ways to understand the themes on a more synthesized level (Borell et al., 2012).

## Findings

First, we present each of the five cases’ journey, from work life before the diagnosis to the present state, ending with a short synthesis of the formal and decisive journey sequences in each case. Thereafter, themes from a joint analysis of the cases are presented together with the synthesized interpretations.

### Simon (case 1)

Simon, 55 years old, had been employed in the same organization in a service profession for 30 years, and he characterized his work life as “great fun” and “very rewarding”. His work-role had developed over the years to leading around 25 co-workers and being responsible for big events with complex logistics. Eventually, when the enterprise downsized a few years earlier, his position was cut, resulting in termination with six months’ notice to him. Looking back, he now viewed himself as partly responsible for making his own position redundant. This happened in the same period as his wife urged him to go to primary care to check his memory even if he himself did not experience any issues. There had in fact been some subtle memory issues a couple of years earlier, according to his wife, but his physician had assured them not to worry. This time he was sent for a specialized memory investigation, where he was diagnosed with young onset AD.

As he got the diagnosis after his employment had been terminated, his wife reported that Simon was already outside the social security system related to employment. In her view, his former director role made him very loyal to the employer; hence, he did not fight for his right to support, even if she tried to push him to put pressure on the company. His physician had recommended him to leave work completely, given the diagnosis, but he still felt he had much to give. Suddenly he had to start looking for a new job, which was a new experience for him after working for the same company for so long. This was a period of uncertainty to both him and his wife. However, he was recruited to a six-month position to lead a project in another company. Simon reported that he disclosed his diagnosis in the middle of those six months, but only to his closest boss, who was surprised because nothing had shown up in his work. He felt he carried out the six months’ employment successfully. He said: “Now I don’t know what the alternative would have been if I had continued and not left (the earlier position) and started the new job (the six months project). Yes, what the consequences would have been if it had shown that I didn’t manage my job, so it felt good somehow to end with the flag up high, a bit like it wasn’t me who had to leave”. Yet, after the six months, Simon discovered that he did not fulfil the revised specifications for the position, so he could not re-apply. Hence that was the end of working life for him.

When we met him, a week after ending the last employment, he explained he was very content with leaving work at a point where he still felt he had made good contributions. He was now looking forward to the luxury of having plenty of time to his disposal, being the same person as before, who was no longer working. He said that working on the final six-month project had strengthened his self-esteem, and he retrospectively pondered that there had not been much potential for personal development in his former director position.

*Synthesis of the formal and decisive journey sequences*: Simon first received sickness benefit, waiting for a decision from SSIA to get sickness compensation. His wife elaborated on the complex insurance situation. As he had lost the safety networks related to permanent employment just before the diagnosis, this left the family in a void of great financial uncertainty. They had to fight with the SSIA without the support of an employer, and they both felt that flexibility was lacking in the SSIA system. Several months after work cessation they were still waiting for SSIA’s decision. This delay also hindered Simon from applying for his retirement pension, as the decision from SSIA had to come first.

### Anna (case 2)

Anna, 60 years old, had been employed by the same tech-company for 30 years, and she described her work-life in very enthusiastic words with variation of work tasks and roles, with opportunities to travel, socialize, and learn. When we met her, she had already left work around six months earlier, but was eager to share how she—with the employer’s tailored support—had been able to continue working 75% of full time for 4 years after receiving a dementia diagnosis.

In contrast to the other cases, Anna’s symptoms did not appear gradually, but her dementia was discovered as a secondary outcome when in hospital for another condition. In her own words, “they discovered that I have a hole in my head”. She reported that the physician strongly recommended sick-leave of at least 50%, but she wanted to continue working. As the prognosis was hard to predict in her case, and she had been with the company for so long, her employer created a tailored work position, which was formally seen as rehabilitation. She stepped down from leadership and took on less complex work tasks appropriate to her current skills. She also worked with a co-worker who was responsible for adhering to timelines and deliverable requirements, e.g., meeting deadlines. A colleague of her own choice was assigned to be her supervisor (included here as one of Anna’s significant others). Both reported that they had weekly digital meetings, catching up on issues or concerns, failures as well as successes, and planning. They had an agreement that the significant other should also be a mediator between Anna and other colleagues involved in her work tasks, openly sharing any feedback on her work performance directly with her. Anna stated: “I had an agreement with them; you must tell my boss if you think I don’t function good enough, and this worked out well for a couple of years—I told them that they have to be able to use me in the best way and trust that I do things right”. Anna openly disclosed the diagnosis and most immediate challenges; she explained she had particular difficulty identifying the persons she currently interacted and worked with, because her workplace was a large activity-based office landscape with no individual desks. Yet, she felt the value of being part of the social community in the workplace outweighed the challenges. Anna’s supervisor and daughter reported that as more issues were raised in her work performance, she was invited to a discussion about the potential fit between her capacity and skills on the one hand, and on the other, the positions available in the company. Anna explained: “I got a long list of available vacant positions, but I could realize that they required English language, among other things … ” From that, she eventually concluded that there were no positions for her, she said “No, I find nothing here that would work in the long run” and she agreed to leave work with six months full salary, even if it was reluctantly, according to her daughter. When we met her, she was satisfied with that choice. In her own words: “one of the important things was that I was given … HR had gathered a list of work positions that were (vacant), I knew they tried to find, and then when I myself concluded that none of these worked … and eh. I realized that there were certain things that … (long pause) well, that worked less well … ”

*Synthesis of the formal and decisive journey sequences*: Anna’s journey went from being well and working full time, to a sudden serious illness with sick-leave. This was followed by continued sick-leave due to dementia, and soon thereafter going back to part-time sickness benefit and part time working with specific tasks and close supervision. Finally, 4 years later, she left work with six month’s salary. After a long wait, she eventually received sickness compensation.

### Lisa (case 3)

Lisa, 53 years old, had been employed for many years by the same company, with complex work-tasks and leadership roles. She had enjoyed socializing with her colleagues but kept her private life to herself, always putting work before her personal life as a single person. As she had worked so hard for so long, it seemed self-evident to her as well as to those around her to interpret her first signs of memory difficulties as symptoms of “burn-out”. In an effort to support her change in abilities, Lisa, her sister, and work leader all described how the company created a position for her with new but very simple work tasks compared to her earlier work. The work leader explained how they designed close, individual support in a safe environment together with co-workers she knew but had not worked with before. This model for return to work had been tailored and successfully used before with other employees suffering from burn-out symptoms, according to her work leader. However, it soon became clear that Lisa could not manage these work tasks, and she was increasingly exhausted just by coming to work. In her own words, she felt increasingly stressed and angry, ashamed, and worthless—she understood that the tasks were “ridiculously simple”, so she could not understand why she still failed. Lisa said: “ … and even if they tried to help me, they should have credit for that … in the workplace and the bosses and all … I have nothing to complain about … still it feels weird, that you are worth nothing (quaver in her voice). Well, I had to, eventually I could not accomplish any work at all—that was when I was asked to go home”. She also felt she was letting her workmates and employer down, even if it was impossible for her to improve her performance.

Lisa, her sister and the representatives from work and rehabilitation all told the same story: the assumed diagnosis of burn-out led to repeated periods of sick-leave and attempts to return-to-work-rehabilitation, eventually also supported by the company’s occupational health care, but the problems she experienced were not solved by this and she did not recover as expected. Consequently, the usual insurance-based plan for return to work by stepwise increase of work hours failed, and Lisa could not speak for herself and explain her difficulties to the SSIA. Hence, the SSIA was ready to withdraw all her financial support. Before such a decision could be made, she went through a specialized memory investigation. Eventually, after more than a year of deteriorating function and periods of sick-leave and failing rehabilitation efforts, this investigation produced a diagnosis of young onset AD. According to Lisa’s sister, the physician said: “you should have come earlier”. To Lisa, the diagnosis was a relief, and when we met her six months after the diagnosis, she was fully occupied with coming to terms with her diagnosis, and with what had happened and what it all meant in terms of grief and feelings of lack of worth.

*Synthesis of the formal and decisive journey sequences*: Lisa’s journey went from subtle difficulties in work tasks, through a long back-and-forth process of sick leave with sickness benefit and adapted work, until eventually she was at risk of losing all insurance support. When her dementia diagnosis was established, the medical assessment was firm; she should not go back to work. She was still on sick leave during the study, and her sister struggled with the state agencies to make sure Lisa got the support she needed and the financial compensation she was entitled to. Lisa also had extensive difficulties in the daily chores at home. For example, she could not manage grocery shopping or cooking without support. Eventually, she was entitled to sickness compensation as well as day care.

### Maria (case 4)

Maria, a 58-year-old administrator, had always worked a lot, but in different fields. A couple of years before we met her, she had started to sense subtle changes, and these worried her as she had seen her mother develop dementia. Hence, she went to a primary care physician, who she reported dismissed her worries twice, suggesting she suffered from symptoms of “burn-out” even though she was at an 80% workload. She felt work demanded all her energy, and finally a specialized memory investigation concluded prodromal AD. This was both a confirmation and a shock to her, and she needed four months sick leave to come to terms with the situation.

When we met her, she worked 40% of full time with customized administrative tasks. Her employer made a choice, she said; instead of just letting her go, which according to her would have been normal considering her diagnosis, the company managed to create a time-limited, adapted, part-time position with simple, routine administrative and filing tasks, yet with much independence, as she wished. In the first interviews, Maria expressed appreciation and gratefulness for this opportunity, and she felt she did a good job even if it was very simple compared to her formal duties. Maria said: “I find it very important to stress here that they were incredibly good to me. They could have said ‘I’m sorry but we have nothing that you are capable of doing here.’ They could have said that, but they did not. But I’m incredibly grateful that I got that possibility, actually. And I also understand that they have looked outside the boxes to make it work … eh … and I, actually, I’m very impressed that they did this. I had not believed that.” She also felt she recovered, so her conclusion was that she probably also had suffered from ’burn-out‘. As her adapted work-tasks did not occupy all her time, she also helped work-mates as needed, and she enjoyed still being part of the work community. She explained she had settled with her diagnosis and planned to work for about one more year.

For Maria, working 40% was the right amount; she needed the rest of her days to manage her condition: “I needed those three days to recover and find myself, to try to do things that would make me feel better”, she said. She planned to retire together with her husband when the employment was projected to end, but she hoped to contribute to a planned new family company together with him. However, in a subsequent interview half a year later when she had left her work as planned, her situation had changed. After a long holiday abroad, the AD diagnosis was confirmed but her cognitive function had improved to a state that made her physician conclude she could have 5–10 years remaining with almost full capacity. While this of course was very good news to Maria, it also caused great frustration as she had exited work life because of the diagnosis. She also started to critically reflect upon how this exit happened. She pondered: “Why was I dismissed when I had not made any mistakes in my work role?” Moreover, much time had been totally devoted to managing—in her view—a scary and devastating diagnosis, searching for help, and even eventually planning to escape, through euthanasia. In addition, much money had been spent on curative products. A few additional months later, she was still enjoying a leisurely life abroad and taking care of herself, and she said: I feel I have got a second chance”. Yet, she felt that the stereotype view of her diagnosis had rushed the employer’s process to end her work life and she was not happy about the way she had been treated. She pondered: “Did I tell you how they reacted at work? That they actually once said they did not trust me anymore? I find it disturbing that they have that view, because this is important for what you (the researcher) do, how one is treated. They expected it (the disease progression) to be much faster, and they were kind to still keep me, but it ended up in such a weird way and I believe that this happens to many others.”

*Synthesis of the formal and decisive journey sequences*: Maria’s first subtle experiences of cognitive decline were initially medically interpreted as “burn out”, before they led to the diagnosis of prodromal AD and four months sick-leave. After this she went back to part time sick-leave and part time work with adapted work tasks and an agreement with the employer to retire within the year. However, after leaving work, Maria’s state had stabilized to almost full capacity causing her to question some of the choices her employer made, and to which she had agreed.

### Carl (case 5)

Carl, 71 years old, reported that he had learned his profession over a long work-life in different positions within the same organization, with continuously increased responsibilities, until retirement at 65. He had always worked a lot, he said, and accepted work had a significant influence on his lifestyle, for example through unhealthy habits and stress. After retirement, he and his wife ran a private consulting enterprise where he worked part time with PR-agencies and as a board member. He reported that about two years before inclusion in this study, his wife noticed that his memory was not as good as before, but his primary care physician twice assured him that he was well. However, the third time the physician sent him to a specialized memory investigation where he was diagnosed with AD. Sometime after that, he and his wife decided to sell their enterprise, but he did not link this to his diagnosis as neither he nor his friends or grown-up children noticed any symptoms; only his wife, he said.

When we met Carl, one year after the AD diagnosis was verified, he worked around 25% of full time as a consultant and board member in another organization, a position he had been recruited to some years ago. He appreciated the monthly work meetings and travel. He felt he could contribute fully in his role and reported that his employer seemed to be very happy and appreciated his work: “They are very glad to have me there, you know, and they do listen a lot to me”, he said. While he experienced some problems in his private life, such as difficulties remembering and finding names in conversations, he had not experienced any incidents in his work role related to his diagnosis. His explanation was that in his work role, he was expected to give immediate feedback on something that was presented to him, but not required to remember details such as names or initiate and organize what was going to happen—hence it worked. His employer verified that Carl contributed fully at work, and the employer reported he had fought for Carl more than once when other board members worried about how dementia might influence Carl’s ability to fulfil his role. The employer’s position was that a diagnosis per se is of no interest if the person contributes. The employer explained: “So this is a bloody nuisance as I see it, people that get a disease are automatically driven out from work life just because they got dementia, the name in itself says ‘out’! Carl is a living proof of ability to work despite having dementia, that is proven, he is absolutely one of the cleverest in the board.” As the COVID-19 pandemic struck in the midst of Carl’s case, his journey came to a stand-still, with him maintaining digital contact with work only occasionally, and also hindering further interviews.

*Synthesis of the formal and decisive journey sequences*: Carl left full-time employment at retirement age but continued working within the family enterprise, though for fewer hours. After his AD-diagnosis, he continued to work for some time until the enterprise was sold. However, Carl still maintained his role as a board member in another organization and had no plans to leave for as long as both he and the employer were content with his contributions.

## Joint analysis of the cases

The joint analysis of the five cases resulted in two intertwined themes: 1) *The significance and consequences of a dementia diagnosis; a double-edged trigger*, and 2) *Sensemaking and agency*. Together, these themes illuminate the features that came to the fore as decisive for how their diagnosis and its consequences were dealt with at work and how the journeys and different routes were experienced, illustrating how such journeys might evolve when a person develops dementia while still engaged in paid work (see Table II). The themes represent a conceptual level, and the sub-themes articulate the key topics underneath (i.e., a more “experience-near” understanding of data). The first theme illustrates how assumptions and expectations in society create conditions that the participants with dementia face and have to navigate, while the second theme illustrates the aspects that seemed most decisive in their process of coming to terms with their condition.

**Table II.** Findings: Overview of themes and subthemes.

Theme	Sub-theme
The significance and consequences of a dementia diagnosis; a double-edged trigger	Who can and cannot get dementia? What is dementia and what will it bring?
Sensemaking and agency	What is happening and how can I have a say?

### The significance and consequences of a diagnosis; a double-edged trigger

In our efforts to understand what happened in each participant’s journey, the commonsense and stereotype view of dementia in health care as well as work life, stood out as a main theme. This view was revealed as decisive assumptions about who can and cannot get dementia, what dementia is and what it will bring. Actions emanating from such assumptions could lead to different consequences: a dementia diagnosis could work as a double-edged trigger, initiating actions leading to very different positive and negative consequences.

#### Who can and cannot get dementia?

The first feature that stood out as significantly contributing to the participants’ experiences was the lengthy processes they had to go through, with a variety of detours, before eventually getting the dementia diagnosis. The main explanation we found for the long delay was that dementia was not even considered an option by the medical experts as the participants were too young and/or functioning too well. In other words: they did not meet the standard profile of a person with dementia. In four cases, the first primary care investigations did not take their complaints seriously, or dismissed their concerns, stating “no problem” (Simon and Carl) or interpreted them as signs of burn out syndrome (Lisa and Maria). This exemplifies the power of the common view of dementia, and of who can and cannot get dementia.

Overall, the delay took place in the first line of health care. Once the participants came to specialized memory investigation the diagnosis was rapidly reached. This long process had particularly serious consequences for one of them (Lisa), but they all experienced living with subtle signs of something not being right while trying to keep up with the usual demands at work and in private life. However, for Lisa, the long process and the misinterpretation of her symptoms, lead to a negative spiral with devastating consequences lasting well after work had ended. These included her being placed at immediate risk of losing her sick-leave salary as she still did not cope and recover as had been expected, due to her condition being interpreted as burn-out.

In contrast, Anna received her dementia diagnosis when she was treated for another serious condition, before symptoms had a major impact on her work performance, hence the process of adjusting work life was initiated at an early stage. Thus, while the other cases had spent a long time coming to terms with subtle symptoms and uncertainty, Anna had been able to spend that time on adaptation and continued work. Together the cases show how sensitive and important the earliest phase might be; from the first experiences of something not being right, to eventually receiving a diagnosis. Yet, our continued analysis also showed how the diagnosis of dementia might trigger unforeseen and unwanted consequences, as well as demand a new orientation in life.

#### What is dementia and what will it bring?

Generally, the participants felt the diagnosis offered explanations to their perceptions of (more or less subtle) changes or symptoms, based upon the commonsense apprehension of what dementia is and what it will bring. Even if the diagnosis was a shock, it also gave something to relate to when looking ahead, a new point of departure. Without a doubt, receiving a diagnosis was a key event and turning point in all five cases, however the results were not always positive. Maria’s experience showed another facet of the diagnostic’s meaning: when her diagnosis had been made, the prognosis was apparently built on the commonplace and stereotyped image of dementia—even if prodromal means very early symptoms. For her employment, this meant that an agreement was developed, stating that she was to exit after a certain time. Although she had managed to do her former work well, she was given very simple work tasks for the remaining period of employment. For her private life, it meant planning for exiting life (through medically-assisted dying) once the expected deterioration set in. When the prognosis suddenly changed to be much more optimistic, she felt robbed of years of her life due to misinformation, with serious consequences in both her work and private lives.

A negative stereotyped view of dementia was also apparent to some extent in Carl’s case: his employer reported resistance among Carl’s fellow board members to trust a colleague with dementia. But in Carl’s case, the conditions differed: the explicit view of his employer—ignoring diagnostic labels as long as employees performed and contributed at work and having a strong advocate in a position of influence in the workplace—allowed Carl to fulfill the work expectations and seemed to support his own view of still maintaining his role. In our understanding, these cases illustrate how a diagnostic label such as dementia might trigger actions that contribute to a variety of life changing consequences—all based upon commonplace perceptions and assumptions of what dementia is and what it will bring.

### Sensemaking and agency

While the participants with dementia shared the same stereotypical view of dementia as the societal stakeholders, they also had to deal with comprehending what happened and navigating the consequences. This theme provided insights into how their opportunities to make sense of and make their voices heard contributed decisively to how they experienced the journey.

#### What is happening and how can I have a say?

The analysis revealed extensive differences between the participants with dementia regarding possibilities to make sense of actions and decisions taken; to have a say and influence what happened in their employment. In our understanding, this turned out to be decisive for how the journey and its consequences were experienced in each case. At a first glance, Anna, Lisa, and Maria were similar in that all three received adapted work tasks. Interestingly, the kind of adaptations were similar, e.g., flexible workhours and simplified tasks, yet they were experienced in different ways and had profoundly different consequences. Anna and Maria were both offered individualized, adapted work tasks after receiving their diagnosis. Both reported these adaptations were a great support, providing them with a retained sense of self-worth, capacity, and agency. Lisa experienced the opposite. While the adaptation for Lisa was carefully planned with flexibility, social interaction, and individual support with comparably very low demands in work tasks that were believed to be easy, she found these tasks to be impossible, but neither she nor her supervisor could understand why. The mentor was even more puzzled when discovering that she had no problem whatsoever in a specific task with procedures that the rest of the team struggled with. The most plausible reason seems to be that, for Lisa, the adapted work was based upon the employer’s earlier experiences of what might support a person with burn-out syndrome to slowly return to work, which also meant expecting recovery. Regardless of whether this approach worked in the past for others, it was not adapted to fit the individual profile of Lisa. This means that this mistaken diagnosis with expected recovery guided the adaptation, and less attention was given to Lisa’s particular prerequisites and experiences, which neither Lisa nor her employer could make sense of.

In hindsight, Maria’s case can also be understood as exemplifying how the diagnostic label might overrule the individual’s particular case. Although Maria felt the adapted work was “silly simple”, she said she did not care. This could also be understood as a change in how she viewed herself after having received the diagnosis. Having labelled herself as “a person with dementia” she accepted the downshift to “silly simple” tasks and was grateful for the opportunity to do them. However, once she realized that her diagnosis did not mean imminent and increasing disability according to the stereotypical understanding of dementia, she felt she had been forced out of work based on the label “dementia” rather than on her work performance.

In contrast, Anna was very content with how her employer had made continued work possible. Eventually, after four years, she was presented with the available work positions and tasks in the company at that time, and she concluded that none were a good match with her own profile. This gave her the possibility to be part of the decision to leave work, and she did so with a sense of acceptance even if it still was a huge step: she understood that for her it was time to leave work. In Simon’s and Carl’s cases, ignoring their doctor’s “order” to leave work immediately when diagnosed with dementia also exemplifies the importance of having a say. Moreover, both had been recruited to their latest work, and in Simon’s case this seemed to boost his readiness to eventually come to terms with work cessation. But having a say was not achievable without support from others. All five participants with dementia received extensive support from family, and their employers also took measures to enable continued work. These examples suggest that sensemaking and having a say were important features that profoundly influenced how the journey was experienced by the participants with dementia, and they also highlight the importance of having agency supported in multiple different ways.

## Discussion

The analysis of these five cases shows how our contemporary society’s common, stereotypical understanding of what dementia is and expectations about what it will bring for the individual, significantly impacts the actions taken when a person develops dementia while still working. Our findings confirm the prevalence of the shared image of dementia in society and how it is usually represented. The first subtle symptoms of early-stage dementia are well known, but often misunderstood if they appear at work, because dementia is so strongly linked to old age and dependency. Hence it is not surprising that most studies have shown that a dementia diagnosis meant the end of work life, rarely preceded by a period of reasonable adjustments (Evans, 2019; McCulloch et al., 2016; Roach & Drummon, 2014; Thomson et al., 2019). In that sense our data provide a unique glimpse of what might take place when adjustments are made to facilitate continued work, albeit they were based on different rationales. Our findings confirm that a diagnosis is an important step in understanding the causes of the employee’s changed performance or challenges at work, but this diagnosis carries with it strong connotations of old age, loss of independence and rapid decline in abilities. This deeply rooted social experience of dementia (Williams et al., 2018) and stigma (Low & Purwaningrum, 2020) can be more disabling than the medical symptoms of dementia. For example, employees may be hesitant to disclose the diagnosis, as Simon was, or being pushed to exit work life regardless of capacity, as Maria experienced. On the other hand, disclosing the diagnosis at work, and the disability that is likely to follow, may help co-workers understand what kind of accommodation and support might be needed. Importantly, our findings suggest that the person with dementia should have a say in how and when disclosure is done at work. There exist methods to support agency among people with dementia in such situations. For example, a solution focused coaching approach was found useful in a case in the Finish part of our research on dementia and work (Heimonen et al., 2022).

Ritchie et al. (2018) concluded that employees with dementia have little control over decisions made regarding their employment, as was also shown in a recent study by our research team (Issakainen et al., 2021). When the medical expertise set the diagnosis, the participants in this study were told to leave work—again reflecting the power of dementia stereotypes of immediate incapacity (Low & Purwaningrum, 2020) - even if this recommendation was not always financially supported by the SSIA. Interestingly, four of the five participants did not follow that recommendation but exercised their agency and managed to continue working, at least for some time. Echoing Williams et al. (2018), they refused to be re-categorized to “someone who could no longer be thought of as a capable worker” (p. 222). However, important prerequisites for claiming one’s right to take part in decision making are that one can make sense of the subtle changes that follow from the first onset of cognitive decline, and that the employer also acknowledges the possibility of dementia being an explanation. Moreover, if the person with dementia cannot make sense of the experienced changes, how could he/she communicate and explain the effects at work and the needs for support to the employer? Dementia is generally considered to bring about lack of awareness, but that is challenged by our findings, as well as earlier studies with people in the very mild stages of dementia (Chaplin & Davidson, 2016; Evans, 2019; Williams et al., 2018; Öhman et al., 2001). Rather, findings suggest that the persons’ experiences of subtle changes are given other explanations by themselves and frequently dismissed by health care because of the commonly held views of what dementia is. This is especially so if the person is still of working age.

On a more general level, our cases extend previous research highlighting the need for increased awareness and education on legal and human rights among employers as well as health care personnel (Ritchie et al., 2018; Stavert et al., 2018). For example, dementia stereotypes appeared to influence physician’s responses in our cases, when their concerns were not initially investigated as cognitive complaints. This aligns with previous research indicating that it can take younger people at least four years to be diagnosed after the onset of symptoms (O’malley et al., 2021). When the diagnosis is finally obtained, our cases suggest that the person might need someone to support their agency on their behalf, by acting and speaking for them when interacting with state agencies, such as the SSIA. But seeking support should not exclude the person with dementia as an agent in the process. Rather our findings underscore how decisive their right to supported agency might be for how they experience their journey, for example through support to exercise autonomy and express preferences (Nedlund & Taghizadeh Larsson, 2016) during this challenging period in life.

In contrast to other studies reporting the experiences of people with the onset of dementia at work (Chaplin & Davidson, 2016; Evans, 2019; Ritchie et al., 2018), the participants in our study mostly received some form of adaptation or accommodation. Common principles for reasonable adjustments at work have been gathered in one review based upon employers’ views (Thomson et al., 2019). These include changing or reducing work hours; simplifying tasks; reducing noise and distraction; using technology as reminders and moving to a less senior role. All these strategies to facilitate continued work were seen in our cases, with varying success. According to Ritchie et al. (2018) assessing the needs and abilities of the individual and drawing on disability management policy will be most successful in supporting continued work. Addressing changes in occupational competence and enabling workforce participation choices are also recommended (Andrew et al., 2018). Importantly, one additional lesson learned from our cases is the importance of allowing time and support for making sense of the diagnosis, and space for agency, both for the employee and the employer.

Moreover, Lisa’s case exemplifies how misinterpretation might put the person at risk of being excluded from the insurance system because work rehabilitation expects improvement of the person’s work performance. This reveals that the current system of work rehabilitation in Sweden does not allow a person’s rehabilitation goal to be maintained with decreasing ability, which might be expected and even inevitable in dementia. Essentially, if we want to enable continued work for employees with conditions such as dementia, who can rarely be expected to search for new, more suitable employment in the competitive labour market, and who are unlikely to achieve improved functioning through rehabilitation, the rules for social insurance compensation in connection to work rehabilitation must be revised.

There is a strong consensus in the literature about the necessity of a person-centred approach when a person develops dementia while still working (Andrew et al., 2018; Ritchie et al., 2018). But what this means is harder to define, particularly as work tasks, roles and environments differ so much. No doubt, dementia brings about disabilities that are likely to influence a person’s ability to work. But as with any disability, this must be seen in its context: work always takes place in social and physical environments, with a multitude of aspects interacting at any given time and situation, including the stereotypical beliefs linked to dementia that are held in society (Low & Purwaningrum, 2020).

### Methodological considerations

One limitation in this work is that three of the five participants with dementia had already left work at the start of the project. We had to accept this because finding potential participants who met the inclusion criteria turned out to be very challenging. Yet, all participants vividly provided their experiences of how the situation evolved, even when this was done retrospectively. We had also intended to search for variation in type of employer and work, but the five cases turned out to work for fairly large enterprises, in white collar or service professions, with a long time in that employment. Thus, we can assume that due to their size, these employers had possibilities to offer adjustments or alternative work tasks that not all employers, including self-employment, might have. In addition, several of our cases involved a long history of employment with one employer, which might have facilitated continued work, as Öhman et al. (2001) reported 20 years ago. However, we still discovered extensive variations in the five cases, and the longitudinal design revealed decisive aspects for the turns that the journey could take and what it might mean to a person.

## Conclusion and implications

The joint analysis exemplifies the power of the biomedical diagnosis of dementia. It served as a mirror altering the reflection of the person, at work and in health care as well as for him-/herself. For all cases, the interpretation of their emerging disability as dementia, plus receiving a diagnosis, were necessary for relevant actions to be taken at work, and for how the process was experienced. On the other hand, the diagnosis and disclosure at work—especially if given in a prodromal stage—could bring about a stereotyped process of exiting work life due to expectations of rapid decline, hence the feeling of being robbed of years of active engagement. This illustrates how the diagnosis of dementia is alien in work-life, but once it is diagnosed, it can trigger self-fulfilling expectations based upon the stereotypical understanding of what it will bring. Having the opportunity to make sense of what is happening and the opportunity to participate in decision-making concerning oneself, contributed decisively to how the journey was experienced by the participants with dementia. Yet, in all cases, other people speaking and acting for the person with dementia were indispensable to support the agency of the person with dementia, regardless of whether it was family, professionals, colleagues or employers, i.e., relational safety nets, as long as advocacy was done with insight, knowledge and respect of the person with dementia’s wishes.

Our findings suggest that key factors for continued work are knowledge and communication between employers and employees with dementia as well as their workmates, significant others, and state agencies. We argue that a shift is needed from a deficit-focused perspective where a stereotyped image of dementia guides all decisions, to a citizenship-approach viewing people with dementia as citizens capable of agency (Boyle, 2014). Such a citizenship-approach could facilitate communication by acknowledging the person with dementia as an agent with the right to self-determination even if he/she might need support to express wishes and make decisions (Nedlund & Taghizadeh Larsson, 2016). Recognizing dementia as a cause of disability represents a rights-based approach, which is more important than ever as long as dementia appears to be an alien in work life, in order to ensure that national provisions apply as equally to people with dementia as to people with other disabilities.
